# Placement-induced effects on high tibial osteotomized construct - biomechanical tests and finite-element analyses

**DOI:** 10.1186/s12891-015-0630-2

**Published:** 2015-09-04

**Authors:** Chu-An Luo, Su-Yang Hwa, Shang-Chih Lin, Chun-Ming Chen, Ching-Shiow Tseng

**Affiliations:** Department of Mechanical Engineering, National Central University, Taoyuan, Taiwan; Graduate Institute of Biomedical Engineering, National Taiwan University of Science and Technology, 43 Keelung Road, Section 4, Taipei, 10607 Taiwan; Department of Orthopedic Surgery, Tri-Service General Hospital, Taipei, Taiwan

**Keywords:** Knee, High tibial osteotomy, Finite-element analysis, Biomechanical test, Tibial plate

## Abstract

**Background:**

High tibial osteotomy (HTO) with a medially opening wedge has been used to treat osteoarthritic knees. However, the osteotomized tibia becomes a highly unstable structure and necessitates the use of plate and screws to stabilize the medial opening and enhance bone healing. A T-shaped plate (e.g. TomoFix) with locking screws has been extensively used as a stabilizer of the HTO wedge. From the biomechanical viewpoint, however, the different plate sites and support bases of the HTO plate should affect the load-transferring path and wedge-stabilizing ability of the HTO construct. This study uses biomechanical tests and finite-element analyses to evaluate the placement- and base-induced effects of the HTO plates on construct performance.

**Methods:**

Test-grade synthetic tibiae are chosen as the standard specimens of the static tests. A medial wedge is created for each specimen and stabilized by three plate variations: hybrid use of T- and I-shaped plates (TIP), anteriorly placed TomoFix (APT), and medially placed TomoFix (MPT). There are five tests for each variation. The failure loads of the three constructs are measured and used as the load references of the fatigue finite-element analysis. The residual life after two hundred thousand cycles is predicted for all variations.

**Results:**

The testing results show no occurrence of implant back-out and breakage under all variations. However, the wedge fracture consistently occurs at the opening tip for the APT and MPT and the medially resected plateau for the TIP, respectively. The testing results reveal that both failure load and wedge stiffness of the TIP are the highest, followed by the MPT, while those of the APT are the least (*P* < 0.05). The fatigue analyses predict comparable values of residual life for the TIP and MPT and the highest value of damage accumulation for the APT. Both experimental and numerical tests show the biomechanical disadvantage of the APT than their counterparts. However, the TIP construct without locking screws shows the highest stress at the plate-screw interfaces.

**Conclusions:**

This study demonstrates the significant effect of placement site and support base on the construct behaviors. The TIP provides a wider base for supporting the HTO wedge even without the use of locking screws, thus significantly enhancing construct stiffness and suppressing wedge fracture. Compared to the APT, the MPT shows performance more comparable to that of the TIP. If a single plate and a smaller incision are considered, the MPT is recommended as the better alternative for stabilizing the medial HTO wedge.

## Background

High tibial osteotomy (HTO) with a medial opening wedge is a surgical treatment for correcting knee osteoarthritis limited to the medial compartment [1–3]. It is more favorable than lateral closing wedge osteotomy due to the complications such as compartment syndrome, neurological complications, lateral muscle detachment, proximal fibula osteotomy, and limb shortening [4–6]. However, the opening wedge can potentially make the proximal tibia highly unstable and necessitates instrumentation by plate and screws to keep the graft in place and stabilize the osteotomized construct [6–8]. Several plate systems have been used for medially opening HTO, such as TomoFix, Puddu, Position HTO plate, and traditional DCP plate [4, 5, 7, 9, 10]. Among them, the TomoFix system is comparatively more commonly used to stabilize the opening wedge by using locking screws [5, 9, 11].

From the biomechanical viewpoint, both implant stress and wedge stability are highly correlated with implant stiffness and placement site [6, 12, 13]. For the T-shaped plate, the anteromedial and medioposterior regions have been selected as the sites of plate placement [6, 14–16]. The placement-induced effects of HTO plates have been investigated in terms of the tibiofemoral loads and the tibial slope [15, 17–19]. Blecha et al. proposed that the contact sites of the tibiofemoral joint occur at the more anterior region for up-right standing with a lower flexion angle [6]. They suggested that the optimal site of the plate placement is at the anteromedial region to provide more stable support [6, 14]. However, Rodner et al. revealed that the anteromedial placement of the HTO plate increases the tibial slope at that region and shifts the tibiofemoral contact to the posterior side. This increases the shearing force on the tibial plateau, which might lead to instability and injury of the soft tissues [15]. Consequently, they recommend that surgeons place the plate at the medioposterior site for better maintenance of the tibial slope [15, 18]. Up to the authors'knowledge, however, detailed comparison of the construct performance between two placements has still not been extensively investigated and the optimal placement remains a controversy. This constitutes the motive of this study.

This study aims to evaluate the placement-induced effects of the TomoFix plate on the construct performance. The hybrid use of the T- and I-shaped plates is applied as the control group to provide a wider base to support the tibiofemoral loads. This is done to eliminate the placement-selecting problem induced by the smaller TomoFix plate. The short- and long-term effects of plate placement are evaluated by biomechanical tests and fatigue simulation. The findings of the current study provide insight into the device- and surgery-related factors associated with better outcomes of HTO fixation.

## Methods

### General study design

The performances of two types of HTO plates are evaluated based on biomechanical tests and numerical analyses. Using the synthetic bones as the standardized specimens, the tests measure the wedge stability to investigate the short-term behaviors of the different tibia-plate constructs. The 50 % failure strength of the APT and MPT constructs is adapted as the applied loads of the fatigue analyses to simulate instrumentation of about three months. The numerical results aim to reveal the long-term responses of construct stress, residual life, and wedge stability.

### Experimental tests

**Fig. 1:**
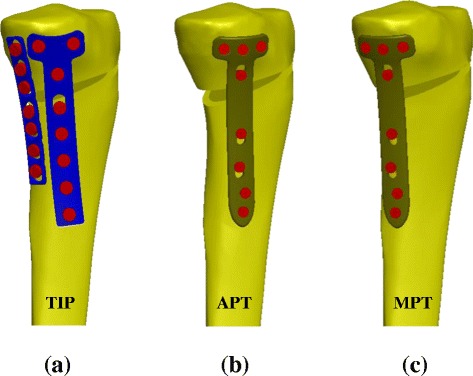
Two types of the high tibial plates are placed at the three locations. **a** Two-leg plate: T and I plate system (TIP). **b** Anteriorly placed TomoFix (APT). **c** Medially placed TomoFix (MPT)

The hybrid use of the T and I plates serves as the comparison baseline of the TomoFix plate (Fig. 1a). The T + I plates are used as the representative of a surgical procedure that can provide wide-base support at the medial side of the osteotomized tibia. Due to the smaller size, there are two possible positions for TomoFix placement can be used to evaluate the placement-induced effects on the construct behaviors (Fig. 1b and c). The symbols TIP, APT, and MPT denote the T + I, anteriorly placed TomoFix, and medially placed TomoFix construct, respectively.

**Fig. 2:**
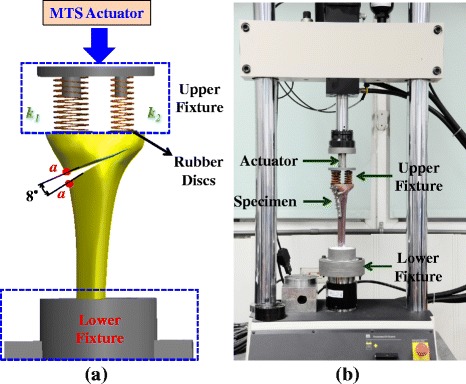
The height and length of the uniplanar open wedge is approximately 9.0 mm and 64.3 mm, respectively. **a** The ratio of the applied loads onto the two plateaus is controlled by the spring constants of the upper fixture. The symbols of the spring constants (*k*_*1*_ and *k*_*2*_) and the points *a* across the opening wedge are defined in the content. **b** The experimental setup of the MTS system, testing specimen, and fixtures

The biomechanical tests are conducted by use of the fourth generation composite tibia (Sawbones Europe, Malmö, Sweden) of which the morphological and mechanical parameters of both cortical and cancellous bones are designed to mimic the human tibia. Only the proximal three-quarters tibia is used as the testing specimen and both plate placement and wedge preparation are determined and created by a senior orthopedic surgeon. The mechanical axis of the tibial specimen is cautiously adjusted coincident with the actuator of the MTS Bionix 858 system (MTS Co., Ltd., Minneapolis, MN, U.S.A.) to simulate the single leg stance. After alignment, the tibial bottom makes contact with the lower fixture and the quick-drying gypsum is used to further stabilize the tibia-fixture construct (Fig. 2). The tibial plateau is removed to ease the mount of two testing springs that the stiffness constants *k*_*1*_ (= 61.25 kg/mm) and *k*_*2*_ (= 40.0 kg/mm) are designed to apply 60 % and 40 % of the knee load onto the medial and lateral sides, respectively. Two rubber discs are separately placed at the spring bottoms to avoid spring subsidence into the cancellous bone (Fig. 2a).

The load-controlled mode is adapted to drive the steady movement of the actuator until construct failure occurs. Construct failure is defined as the visible observation of implant failure (breakage and back-out), bone fracture, and interfacial contact of the wedge surfaces. With a ramp-down waveform, the actuator applies an axial compression at a steady speed of 10 N/sec. The data of the compressive load and axial displacement are respectively measured by loadcell and LVTD sensors with a 50 Hz sampling rate (Fig. 2b). The force-displacement curves of the construct are plotted from the collected data and used to estimate the failure load of the construct. Using a digimatic height gage (Mitutoyo corp., Kawasaki, Japan) as the measurement tool, the wedge micromotion is defined as the distance change between the opposite points *aa* after loading (Fig. 2a). There are five tests for each of the three plate variations. The fracture pattern, wedge stiffness, and opening micromotion are used as the comparison indices of the testing results. The mean and standard deviation of measured results are calculated and statistically analyzed using ANOVA test. The level of significance *α* is chosen as 0.05. The relationships between the experimental and numerical data are analyzed by calculating their coefficients of correlation. The software used to compare the statistical difference is Excel ver. 2010 software (Microsoft Corporation, WA, USA).

### Finite-element analysis

**Fig. 3:**
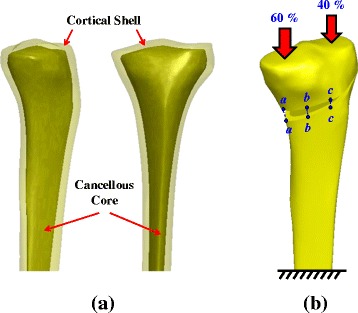
**a** The cortical shell and cancellous core simulated in this study. **b** During actuator movement, the compressive loads onto the medial and lateral plateaus are 60 % and 40 % of the total value, respectively. Three edge lines across the opening wedge are used to measure the height change

Using an experimental specimen as the standard tibia, the fourth generation composite tibia is scanned as CT images with 1-mm slice separation and reconstructed as a tibial model with triangular surface meshes using the software PhysiGuide Ed. 2.3.1 (Pou Yuen Technology Co., LTD, Changhua, Taiwan). The surface model of the proximal tibia is further transformed into a solid model with smooth and seamless surfaces by the software SolidWorks Ed. 2012 (SolidWorks Corporation, Concord, MA, USA). The tibial model consists of a cortical shell and a cancellous core (Fig. 3a). The creation of an opening wedge on the medial side is purposely identical to the tested specimens. No bone graft is inserted into the opening wedge for additional support, thus simulating the worst situation for the HTO plates.

Three medially osteotomized tibiae are instrumented by the TIP, APT, and MPT plates and bone screws in a manner similar to the experimental constructs (Fig. 1). This study omits the screw threads to simulate a rigid-bond situation at the tibia-screw interfaces. The combi-holes of the APT and MPT models are fixed by locking screws. The other plate-screw junctions are consistently inserted with the compression screws. All plates and screws are developed using the software SolidWorks Ed. 2012 (SolidWorks Corporation, Concord, MA, USA). The constitutive laws for the bones (cortical and cancellous) are assumed to be linearly elastic, homogeneous, and isotropic. Young’s modulus and Poisson ration of the tibial sawbones are taken from the literature [20]. The TIP and TomoFix systems are made of 316 L stainless steel and pure titanium, respectively. Except for the locking holes, both tibia-plate and plate-screw interfaces are modeled as surface-to-surface contact elements which allow for separation and slippage. The locking screws are rigidly bonded with the plate holes. The tangential friction law is based on Coulomb’s criterion for delimiting adherence from friction and the friction coefficient is assumed to be 0.3.

The physiological loads are estimated to be 2,000 N (approximately equals to half of the APT and MPT failure strengths) to simulate the compressive load on the knee during single limb stance [21]. In this study, the restoration of the physiological transfer of the knee load is assumed, thus leading to 40 % and 60 % load repartition between the lateral and medial plateau [22, 23] (Fig. 3b). At the tibial bottom, the degree-of-freedom of the nodes is totally fixed. This study uses an automatic mesh generation algorithm with the software Simulation Ed. 2012 (SolidWorks Corporation, Concord, MA, USA), which provides a special element density at the plate-screw junctions three times that of the remainder of the model with an overall average element size of 2 mm. The meshing strategy is designed for curved element boundaries; thus, there are no sharp discontinuities to induce an unrealistically high stress concentration. Using the aspect ratio and Jacobian checks, all elements must be within acceptable distortion limits to maximize the result accuracy. The model is meshed by ten-node tetrahedral solid elements. On average, the final meshes consisted of 105,000 elements and 155,000 nodes for three finite-element models. The similar method has been published in the previous study of the current authors [24].

Under the aforementioned conditions, the single-load analyses of the tibia-plate constructs are conducted to calculate the maximum principal stress of the bone and implants. The fatigue analysis is based on the stress-life method (S-N method). This method predicts the number of cycles to failure by comparing the stress amplitude to a stress versus fatigue life curve (S-N diagram). The alternating stress uses the maximum absolute principal stress calculated from the single-load analysis. The loading ratio is defined as zero to simulate the loading and non-loading condition. For the mean-stress correction, the Gerber and Goodman methods are chosen for 316 L stainless steel and titanium alloy, respectively. Two hundred thousand loading cycles are chosen to simulate the three-month instrumentation [25, 26].

Three numerical indices are chosen to compare the differences in stress distribution, residual life, and wedge micromotion between the three plate variations. The first uses the distribution of the equivalent von Mises stress to show the highly stressed sites of the tibia-plate construct. The second is the residual life of the tibiae and plates, which is used to predict the damage accumulation of the femorotibial loads after three months. The last is the index of construct stability, which is used for measuring the change in wedge height at the edges *aa*, *bb*, and *cc* (Fig. 3b). The convergence test of construct stiffness vs. element number is used to ensure the adequate control of the mesh resolution. The current model is further validated by the stiffness comparison between the numerical and experimental results.

## Results

### Biomechanical tests

**Fig. 4:**
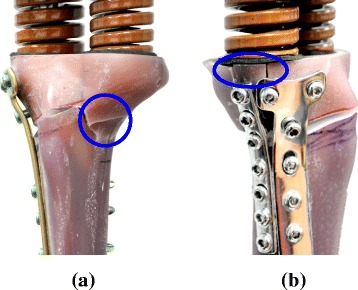
The failure modes of the static tests. **a** TomoFix system. **b** T + I system

Figure 4 shows the typical patterns of the plate variations. All experimental and statistical results of the three variations are listed in Table 1. The fracture sites of all TomoFix constructs occur at the opening tip of the lateral cortex and the wedges collapse (Fig. 4a). However, the fracture sites of the TIP constructs are consistently located at the plateau-plates interfaces and the opening height is significantly higher than that of its counterparts (Fig. 4b). After testing, no plate or screw cracking or back-out can be seen with the naked eye. Even at half (≈4,000 N) of the APT and MPT failure loads (Table 1), the TIP constructs remain intact and no bony and prosthetic failures are observed.

**Table 1:** Testing results of the three plate variations

	Failure load (N)			Wedge stiffness (N/mm)		
	TIP	APT	MPT	TIP	APT	MPT
1	6,584	3,192	4,272	3,076	1,559	1,970
2	6,905	3,506	4,861	3,404	1,776	2,675
3	6,757	3,447	4,638	3,295	1,702	2,459
4	6,936	3,295	4,409	3,682	1,455	2,349
5	6,771	3,623	4,539	3,510	1,987	2,380
Mean ± SD	6,791 ± 140	3,413 ± 171	4,544 ± 224	3,393 ± 228	1,696 ± 205	2,367 ± 256

**Fig. 5:**
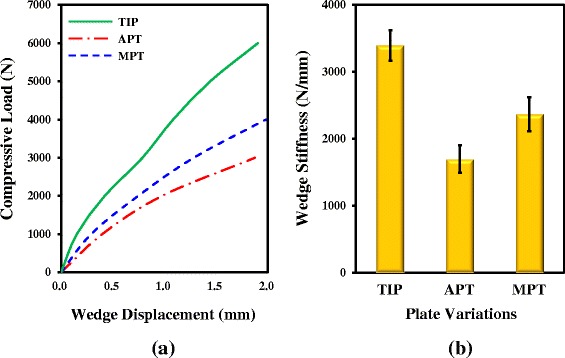
The wedge stability of the three plate variations. **a** The representatives of the three compressive loads vs. wedge displacement curves. **b** The means and standard deviations of the wedge stiffness

The failure loads (wedge stiffness) of the TIP, APT, and MPT are averagely 6,791 N (3,393 N/mm), 3,413 N (1,696 N/mm), and 4,544 N (2,367 N/mm), respectively (Table 1 and Fig. 5). For failure load and wedge stiffness, the ANOVA tests consistently show the statistical significance between the three variation (*P* < 0.05). On averagely, the failure load (wedge stiffness) of the TIP is 99.0 % (100.1 %) and 49.4 % (43.3 %) higher than that of the APT and MPT, respectively.

### Finite-element analyses

**Fig. 6:**
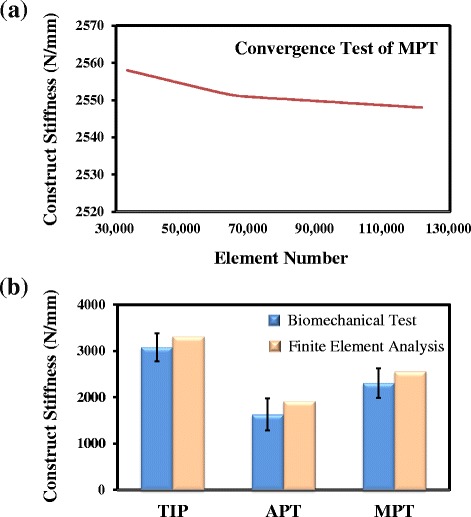
**a** The convergence test of the finite element analysis. **b** The comparison between biomechanical tests and finite element analysis of this study

Figure 6a and b show the validation of the current study in terms of result convergence and construct stiffness. The construct stiffness is defined as the ratio of the entire tibial displacement to the applied loads. During validation of construct stiffness, the instrumented tibiae are subject to the same loads as in the experimental test. The construct stiffness of MPT converges to 2550 N/mm until the element number reached about 105,000 (Fig. 6a). For the convergent stiffness, the errors of the numerical and experimental results are averagely about 7.2 % (Fig. 6b). This indicates that good agreement is achieved and the finite-element model is validated for further analyses.

**Fig. 7:**
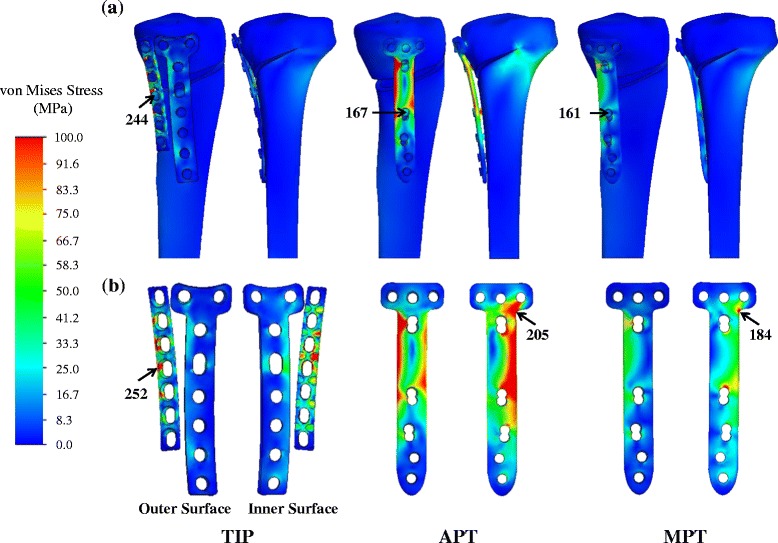
**a** The distribution and maximum values of the von Mises stress for the three constructs. **b** The stress distribution and maximum values of the three plate variations

For the three variations, the von Mises stress distributions of the bone, plate, and screw are shown in Fig. 7. In general, the medial opening makes the implants highly stressed; thus causing some screws and plate holes as the failure potentials for yielding and cracking. The implants of the TIP construct are the most stressed, followed by the APT and MPT. On average, the screw and plate stresses of the TIP construct are 48.9 % and 30.0 % higher than that of the TomoFix counterparts, respectively. For the TIP construct, the I-shaped plate is highly stressed at the holes adjacent to the opening wedge. For the APT construct, the highly stressed volume occurs at the plate corner and the holes around the wedge. Comparatively, the stress-concentrated region of the MPT construct is limited at the plate corner and the hole below the wedge.

**Fig. 8:**
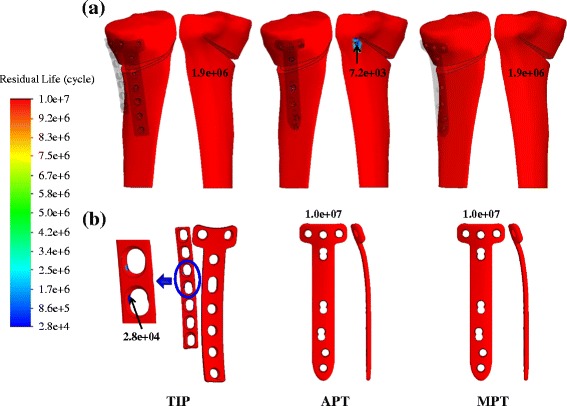
**a** The residual life of the highly osteotomized tibiae. **b** The residual life of the three plate variations

About three months after surgery, the residual life of the tibiae and plates are shown in Fig. 8. Near the wedge tip, the residual life of the APT construct is only 7,200 cycles (Fig. 8a). The fracture site is consistent with the fracture pattern of the APT construct (Fig. 4a). Subjected to dynamic loads from 0 N to 2,000 N, the residual life of the TIP and MPT tibiae are nearly equal. The comparable residual life results of the TIP and MPT constructs are different from both the wedge stiffness and fracture patterns of the static tests, in which the compressive loads are applied beyond 4,000 N. For the TIP construct, the 28,000 cycles of residual life originate at the screw-plate junctions of the I-shaped plate (Fig. 8b). This is significantly less than those of the two TomoFix counterparts. The smaller plate width and the use of nonlocking screws might be contributed to the least residual life of the posterior I plate.

**Fig. 9:**
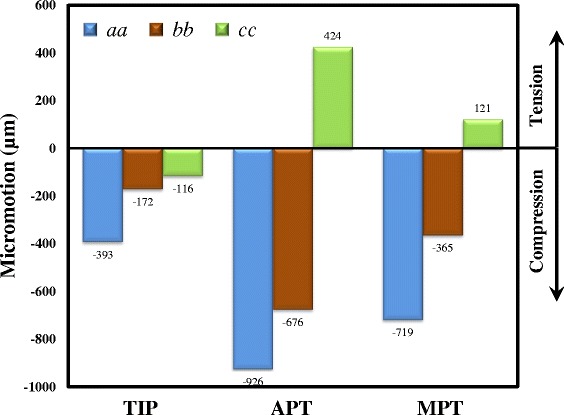
Three wedge micromotions of the three plate variations

The changes in the wedge height of the three edges *aa*, *bb*, and *cc* are shown in Fig. 9. For all constructs, the opening wedges at the edges *aa* and *bb* are consistently compressed. The opening tips (i.e. edge *cc*) of the two TomoFix variations are distracted. However, the opening tip of the TIP construct is still in compression. For all constructs, the micromotion at the edge *aa* is the highest, followed by the edge *bb*, while the edge *cc* is the least. Compared with the APT construct, the micromotions of the TIP and MPT constructs are reduced by 57.6 % and 22.4 % at the edge *aa*, respectively. The aforementioned differences in micromotion reduction for the TIP and MPT are 74.6 % and 46.0 % at the edge *bb*, 72.6 % and 71.5 % at the edge *cc*, respectively.

## Discussion

Compared with the TomoFix system, the TIP can provide two load-transferring legs to transmit the femorotibial loads across the medial wedge (Fig. 1). This study hypothesizes that the two-leg construct has two structural advantages over the one-leg counterparts. The TIP construct can form a wider base to support the opening wedge and avoid surgical hesitance about plate placement. The testing results demonstrate that both the wedge stiffness and failure load of the TIP construct are consistently the highest among the three variations (Fig. 5). Additionally, the two-leg base can behave as a force-couple mechanism to resist the bending loads of the osteotomized tibia [24]. At the edges *aa* and *cc*, the wedges of both APT and MPT constructs are respectively compressed and distracted (Fig. 9). The transformation from compression to distraction shows the bending characteristics of the TomoFix constructs. However, the distraction at the wedge tip is unfavorable to callus formation and even induces wedge fracture (Fig. 4a) [15, 27].

Comparatively, the three edges of the TIP wedge are consistently compressed and more protected, thus effectively suppressing the increase in tibial slope (Fig. 9). Some studies have claimed that increased tibial slope will redistribute tibiofemoral contact pressures posteriorly on the tibial plateau, open the osteotomy anteriorly, and increase anterior tibial translation and subluxation [15, 28, 29]. At the edges *aa* and *cc*, the wedge micromotion of the APT construct is about 2.36 and 3.66 times that of the MPT construct. This indicates the potential risk of deterioration of tibial slope, wedge stiffness, and residual life in the APT tibia (Figs. 5b and 8a). The plateau fracture of the TIP construct can be attributed to the weakened strength of the resected plateau for the sake of spring placement (Fig. 2a).

The screws of the TIP construct are nonlocking fashion in this study. This induces the interfacial slippage at the plate-screw junctions and makes the contact stress highly concentrated [24] (Fig. 7). In turn, the residual life of the TIP implant is significantly less than those of the TomoFix counterparts (Fig. 8). This can be overcome by the use of the plate-screw locking mechanism. However, there are two rigidity-induced concerns about the TIP construct. The minute micromotion of the TIP wedge might be unfavorable to the bony fusion of the opening wedge [30, 31]. Moreover, the stress-shielding effects of the stiffer TIP system might lead to osteoporosis and delay the bony union [32, 33]. These concerns should be further evaluated by the long-term examination of the clinical study.

For the three variations, the relationships between the failure load and wedge stiffness are evaluated by calculating their coefficients of correlation (*R* value). For the TIP, APT, and MPT, the *R* values of the failure load and wedge stiffness are 0.86, 0.91, and 0.94, respectively. It indicates that the higher stability of the construct wedge can effectively improve the construct resistance to bone failure. The highly negative correlation (*R* = −0.99) between the edge *aa* micromotion and wedge stiffness can serve as the well validation between the experimental and numerical results.

This study neglects the plate-bending effect on the biomechanical properties of the various constructs. In the simulative and experimental models, all plates are well pre-bended before fixing them to the tibial model. However, in the technique manual of the AO TomoFix system, the bicortical screw pulls the distally osteotomized segment towards the plate and forces the plate into suspension, creating an elastic preload, which imposes pressure upon the lateral hinge. From the biomechanical viewpoint, however, the current authors presume that the use of bicortical screw to bend the plate potentially alerts the initially distracted height of the medial wedge and exerts the additional shearing force onto the lateral hinge. This might make the lateral hinge at the higher risk of combining the tensile and shearing loads. The pre-bending effect should be confirmed by further studies.

Similar to any model that attempts to simulate femorotibial complexity, there are some limitations and assumptions inherent in this study. After osteotomy, intervention-induced loads originating from the distraction of the remaining intact cortex, medial collateral ligament, and patellar ligament exist. These loads constitute the stabilizing force on the graft [3, 6]. However, the intervention loads are excluded due to the ease of experimental setup. Limited to the available data sources, the femorotibial loads of the degenerative knee are briefly defined in the tests and analyses. Using elastic springs, only compressive loads are assumed to exist in the experimental and numerical evaluations. The deformation of the rubber discs leads to the weaker stiffness of the tested specimen than that of the counterpart in the numerical analysis (Figs. 2a and 5b). Bone remolding is not simulated in this study; thus the rigidity-induced problem of the overstayed and stiffer plate cannot be evaluated *in vivo*.

## Conclusions

Both placement site and support base have significant effects on the construct behaviors. Even without using locking screws, the two-leg TIP forms an effective stabilizer to resist the bending and compressive loads and, thus, significantly enhancing construct stiffness and suppressing wedge fracture. The MPT construct shows performance more comparable to the TIP construct than the APT. Consequently, the TIP construct with locking screws is recommended for the patients with demands of heavy and/or dynamic loads. In the situation of using a single plate, the MPT construct can be an alternative for stabilizing the medial HTO wedge.

## Abbreviations

HTO: High tibial osteotomy
TIP: T- and I-shaped plates
APT: Anteriorly placed TomoFix
MPT: Medially placed TomoFix
